# Carrying Firearms and Suicide Risk Among Adolescents

**DOI:** 10.1001/jamanetworkopen.2026.26924

**Published:** 2026-08-05

**Authors:** Michael A. Lindsey, Jasmin R. Brooks Stephens, Brittney D. Singletary, Taylor C. McGuire, John K. Dixon

**Affiliations:** 1 Silver School of Social Work, New York University, New York, New York; 2 Department of Psychology, University of California, Berkeley, Berkeley; 3 Department of Psychology, Harvard University, Cambridge, Massachusetts; 4 Noxid Group, New York, New York

## Abstract

**Importance:**

Firearms are the most lethal method of suicide across all ages, with case fatality rates exceeding 90%, warranting urgent public health concern. Understanding how carrying of firearms by adolescents and suicide risk vary by race and ethnicity and by stringency of state firearm laws may inform prevention strategies.

**Objective:**

To determine whether the carrying of firearms is associated with suicide attempts among adolescents and whether this association differs by race and ethnicity and by strength of state firearm laws.

**Design, Setting, and Participants:**

This secondary data analysis of a cross-sectional study of the 2023 state-level combined high school Youth Risk Behavior Survey (YRBS) was performed from August 19 to October 26, 2025. Data were collected during the 2023 YRBS administration, fielded between Fall of 2022 and Fall of 2023, with most state surveys conducted during Spring 2023. Participants included adolescents from 15 states who self-reported firearm carrying, suicide attempts, and race and ethnicity. States with greater than 30% missing data on firearm carrying or 100% missing suicide attempt data were excluded. All percentages are survey weighted to reflect the complex sampling design.

**Main Outcomes and Measures:**

The main outcome was self-reported suicide attempt in the past 12 months (any attempt vs no attempt).

**Results:**

A total of 23 232 high school students (mean [SD] age, 15.8 [1.2] years; 50.5% female; 29.8% Hispanic, 9.7% non-Hispanic Black, 46.6% non-Hispanic White, and 13.9% other race or ethnicity) from 15 US states were included in the analysis. Of these, 1161 adolescents (5.0%) reported carrying a firearm and 2236 (9.6%) reported a past-year suicide attempt. Among firearm carriers, 284 (27.4%) reported a suicide attempt compared with 1952 of 22 071 noncarriers (9.4%). After adjustment for race and ethnicity and state firearm law strength, the adjusted odds ratio for suicide attempts among adolescents who carried firearms was 3.48 (95%, CI, 2.59-4.69) (*P* < .001). Each 10-point increase in strength of state firearm law was associated with 6.7% lower odds of suicide attempts (adjusted odds ratio, 0.93 [95% CI, 0.87-0.99]; *P* = .03). The YRBS did not capture means used in attempts; carrying of firearms was associated with increased risk of suicide attempts by any method.

**Conclusions and Relevance:**

In this cross-sectional study of 23 232 high school students from 15 US states, firearm carrying was associated with suicide attempts by any method, independent of sex, grade in school, race and ethnicity, and state firearm laws. The disproportionately high prevalence among Black adolescents who carried firearms raises urgent health equity concerns, underscoring the need for multilevel prevention.

## Key Points

**Question:**

Do associations between carrying firearms and suicide attempts among adolescents differ by race and ethnicity or strength of state firearm laws?

**Findings:**

In this cross-sectional study of 23 232 US high school students from 15 states, carrying a firearm was associated with higher odds of suicide attempts after adjustment for race and ethnicity and state firearm laws (27% vs 9%). Stronger state firearm laws were modestly protective, and non-Hispanic Black adolescents reported the highest suicide attempt prevalence among firearm carriers.

**Meaning:**

These findings suggest that firearm carrying is associated with risk of suicide attempts among adolescents independent of state firearm laws; racial disparities warrant culturally responsive prevention.

## Teaser

This cross-sectional study assesses whether the carrying of firearms is associated with suicide attempts among adolescents and whether this association differs by race and ethnicity and by strength of state firearm laws.

## Introduction

Suicide is the second leading cause of death among adolescents (high school students in grades 9-12, typically aged 14-18 years) in the US.^1^ In 2020, firearms became the leading method of youth suicide, surpassing all other means.^2^ Firearm suicide attempts are highly lethal,^3^ and access to firearms in the home is associated with substantially elevated risk of suicide among youth.^4,5,6^ The transition from suicidal ideation to attempt occurs in less than 1 hour for most individuals, and many youths who attempt suicide have no prior mental health diagnosis.^7,8^ This impulsivity underscores the importance of means restriction: whereas suicide attempts by drug ingestion result in death less than 3% of the time, attempts with firearms are fatal more than 90% of the time.^3^

Ideation-to-action frameworks suggest that the transition from suicidal thoughts to attempts requires not only a desire for death but also acquired capability, including habituation to fear and fearlessness about death.^9,10^ Among adolescents, carrying a firearm may represent both increased access to lethal means during acute crises and heightened capability for suicide, accelerating the transition from ideation to attempts.

Recent trends^11,12,13^ highlight concerning racial and ethnic disparities in firearm suicide among US adolescents. Since 2014, firearm suicide rates have increased by 245% among Black youths and 98% among Latinx youths, compared with a 6% decrease among White youths.^11,12,13^ These shifts signal a departure from prior decades when firearm suicide rates were highest among White youths and underscore the need to examine the intersection of firearm access, state firearm policies, and racial disparities in adolescent suicide risk.

State firearm laws vary substantially in their content and enforcement. Child-access-prevention laws, permit-to-purchase requirements, minimum age possession laws, and safe storage statutes have been associated with lower rates of suicide among youths.^14,15,16,17,18^ However, evidence on whether these protective effects extend equitably across racial and ethnic groups yields mixed findings,^19^ with some studies reporting attenuated benefits for Black and Hispanic youths, potentially reflecting differential enforcement, variation in firearm storage practices, and structural barriers to policy adherence. For example, safe storage laws may have diminished impact where storage norms diverge from legislative requirements, and permit-to-purchase requirements may be less effective for adolescents accessing firearms through social networks rather than legal markets.^20,21,22^

Although firearm availability is a well-established determinant of suicide,^4,5,6^ prior work has rarely examined adolescent firearm carrying, behavior distinct from household access, as a risk factor for suicide attempts. We hypothesized that carrying a firearm would be independently associated with suicide attempts after adjustment for demographic characteristics and state firearm law strength. Using data from 15 states participating in the 2023 Youth Risk Behavior Survey (YRBS),^23^ we assessed this association and explored whether it differed by race and ethnicity or by strength of state firearm laws, recognizing that prior research has had limited ability to detect small to moderate effect modification.

## Methods

We conducted a secondary analysis of the 2023 Centers for Disease Control and Prevention combined high school YRBS dataset. Data were collected during the 2023 YRBS administration, fielded between Fall 2022 and Fall 2023, with most state surveys conducted during Spring 2023.^23^ The 15 states included were Arkansas, Kentucky, Michigan, Mississippi, Montana, Nebraska, Nevada, New Mexico, New York (excluding New York City), North Carolina, Pennsylvania, Tennessee, Texas, Utah, and Virginia, representing 4 US Census regions (South [n = 7], West [n = 4], Midwest [n = 2], and Northeast [n = 2]). Because this study analyzed publicly available, deidentified data, it did not constitute human participants research, and institutional review board approval and informed consent were not required. We followed the Strengthening the Reporting of Observational Studies in Epidemiology (STROBE) reporting guideline for cross-sectional studies.

### Missingness

We excluded 12 states that did not collect firearm carrying data and 1 state that had 100% missing suicide attempt data (eMethods 1 and eFigure in Supplement 1 provide full exclusion details and participant flow). From 28 765 participants across 15 states, complete case analysis yielded 23 232 students. Missing data rates were 1.9% for race and ethnicity, 4.4% for firearm carrying, and 14.3% for suicide attempts (eTable 1 in Supplement 1). Sensitivity analyses using multiple imputation (m = 30) confirmed robustness under missing at random assumptions (eTables 2-4 in Supplement 1 present multiple imputation diagnostics and results across 6 sensitivity methods).

### Measures

#### Outcomes

The primary outcome was suicide attempt, which was assessed using responses to the question “During the past 12 months, how many times did you actually attempt suicide?” We used the dichotomized version of this variable as provided in the YRBS dataset, with responses coded as any attempt (1) vs no attempt (0). The YRBS does not capture the method used in suicide attempts.

#### Exposures

Carrying a firearm was measured using responses to the question “During the past 30 days, on how many days did you carry a gun?” This question excludes carrying firearms for hunting or sport shooting. The dichotomized version of this variable was used, with responses coded as any firearm carrying (1) vs no firearm carrying (0). We noted that the temporal windows for firearm carrying (past 30 days) and suicide attempts (past 12 months) differed.

Strength of state firearm law was measured using Everytown for Gun Safety’s 2023 state firearm policy scores (scale ranges from 0 to 100; higher scores indicate more restrictive laws).^24^ The composite score evaluates approximately 50 policy areas, including background checks, child access prevention, concealed carry, and safe storage requirements (eMethods 2 in Supplement 1 provides full scoring methodology and policy categories). Scores ranged from 3.0 to 83.5 across included states (eTable 5 in Supplement 1). For secondary analyses, states were categorized as having weak (score ≤12) or strong (score ≥35) firearm laws (Table 1).

**Table 1.** Characteristics of the 15 States Included in the Analysis

State	No. of participants^a^	US Census region	Firearm law score^b^	Firearm law category^c^
Arkansas	582	South	3.0	Very weak
Mississippi	434	South	3.0	Very weak
Montana	3182	West	5.0	Very weak
Kentucky	1664	South	9.0	Very weak
Utah	687	West	12.0	Very weak
Texas	877	South	13.5	Weak to moderate
Tennessee	1077	South	16.5	Weak to moderate
North Carolina	1991	South	25.0	Weak to moderate
Nebraska	542	Midwest	25.0	Weak to moderate
Nevada	1211	West	35.0	Moderate to strong
Michigan	1899	Midwest	35.0	Moderate to strong
Pennsylvania	1825	Northeast	40.0	Moderate to strong
New Mexico	4997	West	40.5	Moderate to strong
Virginia	1776	South	49.0	Moderate to strong
New York^d^	488	Northeast	83.5	Very strong
All	23 232	NA	3.0-83.5	NA

Abbreviation: NA, not applicable.

^a^ Unweighted counts after complete case analysis.

^b^ Calculated using the Everytown for Gun Safety 2023 firearm law rankings.^24^ The scale ranged from 0 to 100, with higher scores indicating stricter policy.

^c^ Categories were defined according to firearm law scores (≤12 = very weak; 13-34 = weak to moderate; 35-49 = moderate to strong; ≥50 = very strong).

^d^ Except New York City.

#### Covariates

Race and ethnicity was self-reported by adolescents and categorized into the following groups: Hispanic, non-Hispanic Black (reference group), non-Hispanic White, and other (including American Indian or Alaska Native, Asian, Native Hawaiian or other Pacific Islander, and multiracial). Sex was self-reported as male or female. Grade in school was self-reported as grade 9 through grade 12.

### Statistical Analysis

Data were analyzed from August 19 to October 26, 2025. All analyses used R, version 4.5.1, with the survey package, version 4.4.2 (R Project for Statistical Computing). Schools served as primary sampling units within strata, with state-specific sampling weights (eMethods 3 and eTable 6 in Supplement 1 provide the full survey design specification, including variance estimation and design effects). States function as independent sampling frames; primary sampling units nest within states, inherently accounting for both school- and state-level clustering. State-level variation was captured through the firearm law score variable, with state fixed effects as sensitivity analysis (eMethods 4 and eTable 7 in Supplement 1 shows <1% change in primary odds ratio [OR]). We used survey-weighted logistic regression with quasibinomial family to account for overdispersion.

Primary models adjusted for race and ethnicity and state firearm law score; sensitivity analyses additionally adjusted for sex and grade in school. A directed acyclic graph guided covariate selection (eMethods 5 in Supplement 1 provides the full confounding rationale, including the directed acyclic graph and confounder vs mediator distinctions). Variables such as depression, substance use, and bullying were not included because they likely function as mediators; adjusting for mediators would underestimate the total effect.^25^ The YRBS does not include individual-level socioeconomic measures.

The primary analyses examined (1) associations between firearm carrying and suicide attempts, and (2) whether race and ethnicity or strength of state firearm laws moderated this association. Effect modification was assessed using multiplicative interaction terms and interpreted on the basis of the width and position of the resulting 95% CIs (eTable 8 in Supplement 1). Secondary analyses examined race-stratified patterns and compared outcomes in states with the weakest vs strongest firearm laws. All analyses used 2-sided α = .05 to indicate statistical significance. Results are presented as ORs with 95% CIs.

## Results

### Study Population

The final analytic sample included 23 232 high school students from 15 states; 11 886 students (50.5%) were female and 11 241 (49.0%) were male, and the mean (SD) age was 15.8 (1.2) years (Table 2). In terms of race and ethnicity, 6929 students (29.8%) were Hispanic, 2262 (9.7%) were non-Hispanic Black, 10 815 (46.6%) were non-Hispanic White, and 3226 (13.9%) were of other race or ethnicity. Among these students, 1161 (5.0%) reported carrying a firearm in the past 30 days (compared with 22 071 [95.0%] who did not) and 2236 (9.6%) reported attempting suicide in the past year. Firearm carriers were predominantly male (889 [74.7%] vs 10 352 [47.9%] of noncarriers; *P* < .001) (eTable 9 in Supplement 1).

**Table 2.** Participant Characteristics by Firearm Carrying Status

Characteristic	Participant group, unweighted No. (survey weighted %)			*P* value^a^
	Total (N = 23 232)	Carriers (n = 1161)	Noncarriers (n = 22 071)	
Age, mean (SD), y	15.8 (1.2)	16.1 (1.2)	15.8 (1.2)	.08
Sex^b^				
Female	11 886 (50.5)	262 (24.4)	11 624 (51.6)	<.001
Male	11 241 (49.0)	889 (74.7)	10 352 (47.9)	
Grade in school^c^				
9	6801 (27.5)	354 (26.9)	6447 (27.5)	.64
10	6840 (25.9)	300 (25.6)	6540 (25.9)	
11	5178 (23.6)	249 (21.2)	4929 (23.7)	
12	4264 (23.0)	225 (26.4)	4039 (22.9)	
Race and ethnicity				
Hispanic	6929 (29.8)	361 (33.3)	6568 (28.1)	.25
Non-Hispanic Black	2262 (9.7)	108 (15.5)	2154 (13.6)	
Non-Hispanic White	10 815 (46.6)	487 (43.3)	10 328 (49.7)	
Other^d^	3226 (13.9)	205 (7.9)	3021 (8.7)	
Suicide attempt	2236 (9.6)	284 (27.4)	1952 (9.4)	<.001
Prevalence ratio (95% CI)	NA	2.91 (2.31-3.65)	1 [Reference]	NA

Abbreviation: NA, not applicable.

^a^ Calculated from survey-weighted χ^2^ tests (categorical) or independent-samples (unpaired) t tests (continuous).

^b^ Data were missing for 105 students.

^c^ Data were missing for 149 students.

^d^ Includes American Indian or Alaska Native, Asian, Native Hawaiian or Other Pacific Islander, and multiracial.

### Firearm Carrying and Suicide Attempts

Among 1161 firearm carriers, 284 (27.4%) reported a suicide attempt, compared with 1952 of 22 071 noncarriers (9.4%), yielding a prevalence ratio of 2.91 (95% CI, 2.31-3.65). In the multivariable logistic regression model adjusting for race and ethnicity and state firearm law score, the adjusted OR for suicide attempts among adolescents who carried firearms vs noncarriers was 3.48 (95% CI, 2.59-4.69) (*P* < .001) (Table 3). Compared with non-Hispanic Black adolescents, Hispanic (OR, 0.62 [95% CI, 0.46-0.82]) (*P* < .001) and non-Hispanic White (OR, 0.53 [95% CI, 0.40-0.71]) (*P* < .001) adolescents had lower odds of suicide attempts; the odds for adolescents of other race or ethnicity was not statistically significant (OR, 0.78 [95% CI, 0.53-1.16]; *P* = .22).

**Table 3.** Multivariable Logistic Regression of Firearm Carrying and Suicide Attempts

Variable	Model, OR (95% CI)^a^		
	Unadjusted	Adjusted for race and ethnicity and score	Adjusted race and ethnicity, sex, and grade in school
Firearm carrying	3.62 (2.68-4.90)	3.48 (2.59-4.69)	4.42 (3.13-6.24)
Race and ethnicity			
Hispanic	NA	0.62 (0.46-0.82)	0.64 (0.49-0.82)
Non-Hispanic Black	NA	1 [Reference]	1 [Reference]
Non-Hispanic White	NA	0.53 (0.40-0.71)	0.54 (0.40-0.72)
Other^b^	NA	0.78 (0.53-1.16)	0.77 (0.52-1.13)
State gun law score (per 10 points)	NA	0.93 (0.87-0.99)	NA
Female vs male sex^c^	NA	NA	0.40 (0.33-0.48)
Grade in school			
9	NA	1 [Reference]	1 [Reference]
10	NA	NA	0.72 (0.56-0.92)
11	NA	NA	0.90 (0.61-1.31)
12	NA	NA	0.78 (0.53-1.13)
No. of participants	23 232	23 232	22 988^d^

Abbreviations: NA, not applicable; OR, odds ratio.

^a^ All models use survey-weighted logistic regression with quasibinomial family.

^b^ Includes American Indian or Alaska Native, Asian, Native Hawaiian or Other Pacific Islander, and multiracial.

^c^ Reflects coding in the Youth Risk Behavior Survey dataset.

^d^ Data for grade in school were missing for 149 students.

Multiplicative interactions between carrying a firearm and race and ethnicity were small and statistically nonsignificant, but with wide 95% CIs that did not exclude clinically meaningful effect modification in either direction (firearm × Hispanic interaction OR, 1.38 [95% CI, 0.56-3.38]; *P* = .47; firearm × non-Hispanic White interaction OR, 0.89 [95% CI, 0.39-2.05]; *P* = .78; firearm × other race interaction OR, 1.00 [95% CI, 0.43-2.32]; *P* > .99). Likewise, neither the firearm × state law score interaction (OR per 10-point increase, 1.02 [95% CI, 1.00-1.04]; *P* = .11) nor the firearm × weak-vs-strong-law interaction (OR per 10-point increase, 1.13 [95% CI, 0.60-2.12]) (*P* = .70) provided evidence that state laws attenuate the individual-level association between carrying a firearm and suicide attempts. Given the width of these intervals, our data are compatible with a range of true effect modification magnitudes; we therefore characterize these findings as inconclusive rather than as evidence of no effect modification (eTable 8 in Supplement 1).

### State Firearm Law Strength

State firearm law scores ranged from 3.0 (Mississippi and Arkansas) to 83.5 (New York), with 5 states classified as very weak (score ≤12), 4 as weak to moderate (13-34), 5 as moderate to strong (35-49), and 1 as very strong (≥50) (Table 1 and eTable 10 in Supplement 1). Each 10-point increase in state firearm law strength was associated with 6.7% lower odds of suicide attempts (adjusted OR, 0.93 [95% CI, 0.87-0.99]) (*P* = .03). Firearm carrying prevalence was higher in states with weak laws (7.0%) compared with states with strong laws (3.9%) (eTable 11 in Supplement 1). In thanalysis comparing adolescents in states with the weakest (n = 6549) vs strongest (n = 12 196) firearm laws, carrying a firearm remained associated with suicide attempts (OR, 4.83 [95% CI, 3.13-7.46]) (*P* < .001). Adolescents in states with strong firearm laws had 22% lower odds of suicide attempts compared with those in weak firearm law states (OR, 0.78 [95% CI, 0.62-0.98]) (*P* = .04). The interaction between firearm carrying and state firearm law category was not significant (OR, 1.13 [95% CI, 0.60-2.12]) (*P* = .70).

### Racial and Ethnic Patterns

Firearm carrying was associated with higher prevalence of suicide attempts across all racial and ethnic groups (Table 4 and eTable 12 in Supplement 1). Among non-Hispanic Black students, 44 of 108 firearm carriers (35.2%) reported suicide attempts compared with 255 of 2154 noncarriers (14.2%; risk ratio [RR], 2.49 [95% CI, 1.49-4.15]). Similar patterns were observed among Hispanic students (95 of 361 carriers [30.5%] vs 554 of 6568 noncarriers [9.2%]; RR, 3.31 [95% CI, 2.05-5.35]), non-Hispanic White students (89 of 487 carriers [21.3%] vs 819 of 10 328 noncarriers [7.9%]; RR, 2.68 [95% CI, 1.92-3.75]), and students of other race or ethnicity (56 of 205 carriers [30.9%] vs 324 of 3021 noncarriers [11.1%]; RR, 2.78 [95% CI, 1.79-4.33]). Non-Hispanic Black students who carried firearms had the highest suicide attempt prevalence of any subgroup.

**Table 4.** Race-Stratified Analysis of Firearm Carrying and Suicide Attempts

Race and ethnicity	No. of carriers	No. of noncarriers	Suicide attempts, No./total No. (%)^a^			OR (95% CI)^c^
			Carriers	Noncarriers	Risk ratio^b^	
Hispanic	361	6568	95/361 (30.5)	554/6568 (9.2)	3.31 (2.05-5.35)	4.33 (2.25-8.36)
Non-Hispanic Black	108	2154	44/108 (35.2)	255/2154 (14.2)	2.49 (1.49-4.15)	3.30 (1.56-6.95)
Non-Hispanic White	487	10 328	89/487 (21.3)	819/10 328 (7.9)	2.68 (1.92-3.75)	3.14 (2.02-4.88)
Other^d^	205	3021	56/205 (30.9)	324/3021 (11.1)	2.78 (1.79-4.33)	3.58 (2.15-5.97)
Overall	1161	22 071	284/1161 (27.4)	1952/22 071 (9.4)	2.91 (2.31-3.65)	3.62 (2.68-4.90)

Abbreviation: OR, odds ratio.

^a^ Percentages are survey-weighted.

^b^ Calculated using survey-weighted prevalences with delta method standard errors.

^c^ Calculated from survey-weighted logistic regression.

^d^ Includes American Indian or Alaska Native, Asian, Native Hawaiian or Other Pacific Islander, and multiracial.

### Sensitivity Analyses

Sensitivity analyses addressing differential missingness confirmed the robustness of our findings. Across multiple imputation (OR, 3.45 [95% CI, 2.60–4.59]), best-case scenario (OR, 3.08 [95% CI, 2.34–4.04]), worst-case scenario (OR, 2.66 [95% CI, 2.05–3.45]), inverse probability weighting (OR, 3.49 [95% CI, 2.57–4.74]), and low-missingness state subsets (ORs, 3.07 [95% CI, 2.16–4.36] to 4.44 [95% CI, 2.57–7.67]), the association between firearm carrying and suicide attempts remained (*P* < .001 for all) (eTable 4 in Supplement 1). State fixed effects sensitivity analysis showed that the association between firearm carrying and suicide remained virtually unchanged (OR, 3.51 [95% CI, 2.57-4.79] vs 3.48 [95% CI, 2.59-4.69]; difference of 0.9%), indicating robustness to unmeasured state-level confounding (eTable 7 in Supplement 1). Adding sex and grade in school yielded a change from baseline of 36% (OR, 3.25 [95% CI, 2.38-4.45] to 4.42 [95% CI, 3.13-6.24]) (eTable 13 in Supplement 1), consistent with negative confounding. The Figure displays ORs across subgroups and sensitivity analyses.

**Figure.**
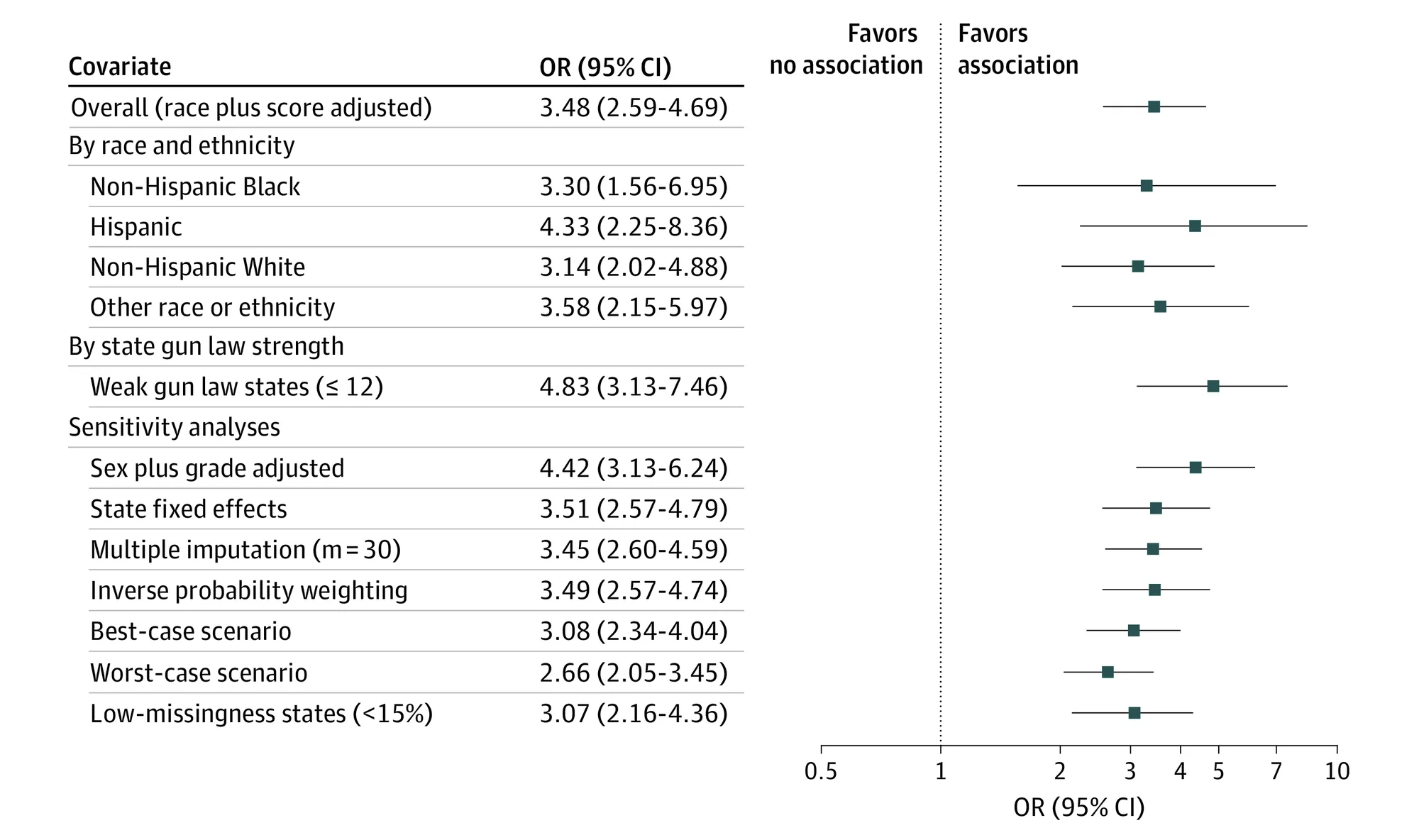
Forest Plot of Association Between Firearm Carrying and Suicide Attempts Odds ratios (ORs) are shown across subgroups and sensitivity analyses. All estimates exceed 1.00, indicating consistent positive associations across subgroups and analytic approaches. The x-axis is on a logarithmic scale.

## Discussion

In this cross-sectional study of more than 23 000 high school students from 15 states, firearm carrying was associated with suicide attempts, independent of race and ethnicity and state firearm policy environment. This association persisted across multiple model specifications and sensitivity analyses, including after adjustment for sex and grade in school, indicating that demographic factors suppress rather than explain the association. Stronger state firearm laws were modestly protective. Non-Hispanic Black adolescents reported the highest suicide attempt prevalence, particularly among firearm carriers. Firearm-related adolescent suicide deaths declined between 1981 and 2015 before sharply reversing,^16,26,27,28,29^ making the current findings particularly urgent.

Our results align with ideation-to-action frameworks,^9,10,30,31^ which argue that acquired capability enables the transition from suicidal thoughts to attempts. Importantly, the YRBS does not capture the means used in suicide attempts, and firearm carriers likely attempted suicide by other methods, consistent with the high survival rate of respondents. Firearm carrying may confer suicide risk not primarily as the method of attempt but because it (1) provides immediate access to lethal means during acute crises, (2) serves as an indicator of broader risk-taking and externalizing behaviors, and (3) signals heightened exposure to violence and trauma. The robustness of the association across demographic groups and policy environments suggests that firearm carrying is a salient marker of risk.^32^

Consistent with prior evaluations,^33^ stronger state laws were associated with reduced suicide attempts, but the effect was modest, likely reflecting heterogeneity in implementation and the fact that most laws do not directly target adolescent firearm carrying. Policies that interrupt weapon carrying (eg, safe storage, community-based violence interruption) and reduce violence exposure may dampen the capability pathway.^34,35,36,37^

Non-Hispanic Black adolescents had the highest prevalence of suicide attempts, particularly among firearm carriers, consistent with documented rising suicide attempts among Black youths.^38,39^ Recent data reveal sex-specific patterns: American Indian or Alaska Native males aged 10 to 19 years have the highest firearm suicide rates, while Black females have experienced the steepest increases.^12^ Structural determinants including residential segregation, concentrated poverty, differential policing, neighborhood violence exposure, and unequal access to mental health services, shape both firearm access patterns and suicide risk among youths from racial minority groups.^40,41,42^ The disproportionate prevalence of firearm carrying among Black adolescents may reflect structural disadvantage and adaptive responses to unsafe environments rather than individual risk preferences, as youths in high-violence neighborhoods may carry firearms for perceived protection.^43,44^ Notably, Black and male adolescents were more likely to report suicide attempts without preceding ideation, complicating detection.^32^ These disparities highlight the need for prevention that addresses structural determinants and culturally specific risk pathways.

### Clinical and Policy Implications

Firearm carrying should be incorporated into routine screening for suicide risk in pediatric and school-based settings, alongside lethal means counseling for families in firearm-owning households. Evidence-based programs include Counseling on Access to Lethal Means (CALM),^45^ the ASK Campaign (Asking Saves Kids),^46,47^ and the Safety Planning Intervention.^48,49^ Implementation partnerships among schools, pediatric settings, and community-based violence interruption programs may strengthen identification and reduce youth firearm carrying. At the policy level, comprehensive firearm legislation including child access prevention, safe storage, and age restrictions remains critical, with emphasis on provisions that specifically reduce adolescent access to firearms.^50^ To address disparities, interventions must address structural drivers, including violence exposure, economic precarity, and unequal access to mental health care that shape both firearm access and suicide capability for racially minoritized youth.

### Strengths and Limitations

Strengths of this study include the large, diverse sample, validated YRBS measures, robust survey-weighted methods (eMethods 3 in Supplement 1), and extensive sensitivity analyses including multiple imputation and state fixed effects (eTables 1, 4, 7, and 13 in the Supplement 1). However, this study has some limitations. Given the cross-sectional design, all findings represent associations and cannot establish causal relationships. The temporal mismatch between firearm carrying (past 30 days) and suicide attempts (past 12 months) precludes establishing temporal sequence. Both YRBS items capture prevalent rather than incident behaviors, and prior research suggests firearm carrying among adolescents reflects a relatively stable behavioral pattern rather than a single episode. Nevertheless, if carrying follows rather than precedes suicidal behavior (eg, if adolescents acquire firearms after a crisis period), the cross-sectional association could overestimate the causal effect. Data were self-reported; social desirability bias would likely lead to underreporting of both behaviors, attenuating the observed association. Multiple imputation confirmed robustness despite 14.3% missing suicide attempt data (eTable 4 in Supplement 1).

Covariate adjustment was limited to demographic characteristics to estimate the total association; variables such as depression and substance use likely function as mediators, and their inclusion would attenuate the estimate (eMethods 5 in Supplement 1 provides a directed acyclic graph and full confounding rationale). Unmeasured socioeconomic confounders could change the association, although state fixed effects showed minimal change (<1%) (eTable 7 in Supplement 1) and the association remained after adjustment for sex and grade in school (eTable 13 in Supplement 1). Data were drawn from 15 states overrepresenting the South and weaker firearm law states, potentially limiting generalizability (eTable 5 in Supplement 1). The composite firearm law score does not permit analysis of specific law components.

## Conclusions

In this cross-sectional study of 23 232 high school students from 15 US states, adolescent firearm carrying was associated with suicide attempts by any method, independent of sex, grade in school, race and ethnicity, and state policy environment, although residual confounding from unmeasured socioeconomic factors cannot be excluded. State firearm laws were modestly protective at the population level; tests for effect modification by race and ethnicity and strength of state firearm laws were inconclusive, with 95% CIs wide enough to be compatible with both meaningful protection and meaningful harm. The disproportionately high prevalence of suicide attempts among Black adolescents who carry firearms raises urgent health equity concerns. Comprehensive strategies—combining restrictive firearm policies, clinical screening and lethal means counseling, school-based prevention, and culturally tailored, disparity-focused interventions—are essential to address the escalating crisis of adolescent firearm suicide.

## Notes

Supplement 1. **eMethods 1.** Missing Data and Multiple Imputation **eMethods 2.** Everytown Gun Law Strength Score Methodology **eMethods 3.** Survey Design and Weighting **eMethods 4.** Ecological Policy Variable Treatment **eMethods 5.** Confounding Control Strategy **eTable 1.** Missing Data Patterns and Characteristics **eTable 2.** Multiple Imputation Diagnostics Summary **eTable 3.** Multiple Imputation Model Specification **eTable 4.** Sensitivity Analysis: Complete Case vs Multiple Imputation **eTable 5.** Characteristics of the 15 States Included in Analysis **eTable 6.** Design Effects and Effective Sample Sizes **eTable 7.** Sensitivity Analysis: State Fixed Effects vs Ecological Variable **eTable 8.** Multiplicative Interaction Terms Between Gun Carrying and Race and Ethnicity and State Gun Law Strength **eTable 9.** Sex-Stratified Gun Carrying and Suicide Attempt Prevalence **eTable 10.** Characteristics by Gun Law Strength Category **eTable 11.** State-Level Weighted Prevalences **eTable 12.** Race-Stratified Cell Counts and Suicide Attempt Prevalences **eTable 13.** Sensitivity Analysis: Demographic Confounding (Sex and Grade) **eFigure.** Participant Flow Diagram (CONSORT Style) **eResults.** Summary of Supplementary Findings
eReferences

Supplement 2.
Data Sharing Statement
