# Errors in Results, Figure, and Tables

**DOI:** 10.1001/jamanetworkopen.2026.35871

**Published:** 2026-08-31

In the Original Investigation titled “Carrying Firearms and Suicide Risk Among Adolescents,” published August 5, 2026, the Results included an incorrect 95% CI for the state fixed effects sensitivity analysis; the odds ratios and 95% CIs in the Figure were incorrect for the overall cohort and for all sensitivity analyses; the title of Table 2 was incorrect; and the odds ratios and 95% CIs in Table 4 were incorrect. This article has been corrected.^1^
