# Supplementary material for: Carrying Firearms and Suicide Risk Among Adolescents
Source: JAMA Netw Open. 2026 Aug 5;9(8):e2626924. doi: 10.1001/jamanetworkopen.2026.26924 (PMC13444804; doi:10.1001/jamanetworkopen.2026.26924)
Supplement: Supplementary file 1 [file jamanetwopen-e2626924-s001.pdf]

Supplementary Online Content

Lindsey MA, Brooks Stephens JR, Singletary B, McGuire TC, Dixon JK. Carrying of firearms and suicide risk among adolescents. *JAMA Netw Open.* 2026;9(8):e2626924. doi:10.1001/jamanetworkopen.2026.26924

eMethods 1. Missing Data and Multiple Imputation
eMethods 2. Everytown Gun Law Strength Score Methodology
eMethods 3. Survey Design and Weighting
eMethods 4. Ecological Policy Variable Treatment
eMethods 5. Confounding Control Strategy
eTable 1. Missing Data Patterns and Characteristics
eTable 2. Multiple Imputation Diagnostics Summary
eTable 3. Multiple Imputation Model Specification
eTable 4. Sensitivity Analysis: Complete Case vs Multiple Imputation
eTable 5. Characteristics of the 15 States Included in Analysis
eTable 6. Design Effects and Effective Sample Sizes
eTable 7. Sensitivity Analysis: State Fixed Effects vs Ecological Variable
eTable 8. Multiplicative Interaction Terms Between Gun Carrying and Race and Ethnicity and State Gun Law Strength
eTable 9. Sex-Stratified Gun Carrying and Suicide Attempt Prevalence
eTable 10. Characteristics by Gun Law Strength Category
eTable 11. State-Level Weighted Prevalences
eTable 12. Race-Stratified Cell Counts and Suicide Attempt Prevalences
eTable 13. Sensitivity Analysis: Demographic Confounding (Sex and Grade)
eFigure. Participant Flow Diagram (CONSORT Style)
eResults. Summary of Supplementary Findings
eReferences

This supplementary material has been provided by the authors to give readers additional information about their work.

© 2026 Lindsey MA et al. *JAMA Network Open*.

Supplement

eMethods 1. Missing Data and Multiple Imputation

Little's MCAR test rejected the assumption that data were missing completely at random (chi-squared = 1210.12, df = 58, P < .001). Pattern analysis showed missingness associated with sex and grade, consistent with missing at random (MAR) conditional on observed covariates. Given modest missingness (19.2 percent excluded) and patterns supporting MAR, we conducted complete case analysis as our primary approach. Survey weights account for unit non-response within the YRBS sampling design.

We performed sensitivity analysis using multiple imputation (mice package v3.18.0, m = 30 datasets) with variable-specific methods: logistic regression for binary variables (suicide attempt, gun carrying, sex), polytomous regression for race, proportional odds models for grade, and predictive mean matching for age. Survey design variables (PSU, strata, weights) were included as predictors but not imputed. All variables converged within 15 iterations. Imputed distributions matched observed distributions (differences < 0.2 percent). Results were pooled using Rubin's rules with Barnard-Rubin degrees of freedom adjustment. Fraction of missing information ranged from 0.18 to 0.25 (moderate influence). Additional sensitivity analyses (best/worst-case scenarios, inverse probability weighting, low-missingness subsets) yielded consistent results.

eMethods 2. Everytown Gun Law Strength Score Methodology

The Everytown for Gun Safety 2023 Gun Law Strength Score evaluates states across approximately 50 policy areas organized into five major categories: (1) Keep Guns Out of the Wrong Hands (background checks, purchase permits, extreme risk protection orders, reporting of prohibited persons); (2) Protect Children and Families (child access prevention laws, domestic violence restraining order firearm restrictions, safe storage requirements, minimum age requirements); (3) Ensure Responsible Gun Ownership (concealed carry permitting, open carry restrictions, firearm registration, waiting periods); (4) Stand Against Shoot First Laws (stand your ground law restrictions, duty to retreat provisions); and (5) Fund Violence Intervention and Community Programs (state-level violence intervention funding, research funding). States receive points for each enacted policy, producing a composite score from 0 (no policies) to 100 (all evaluated policies enacted). The scoring methodology has been used in peer-reviewed research examining the association between state firearm policy environments and firearm mortality outcomes (Everytown for Gun Safety Support Fund, 2023). The full scorecard methodology and individual state scores are publicly accessible at https://everytownresearch.org/rankings/. Limitations of the composite approach include inability to assess the independent contribution of specific policy components and the assumption that all evaluated policies contribute equally to the overall score.

eMethods 3. Survey Design and Weighting

Unless stated otherwise, sample sizes (N) are unweighted counts; percentages and prevalences are survey-weighted; standard errors and confidence intervals use Taylor series linearization; all analyses used R version 4.5.1 and the survey package version 4.4.2.

The YRBS employs multi-stage cluster sampling with schools as primary sampling units (PSUs). We implemented the complex survey design using the survey package (version 4.4.2) in R (version 4.5.1):

© 2026 Lindsey MA et al. *JAMA Network Open*.

svydesign(id = ~PSU, strata = ~stratum, weights = ~weight, nest = TRUE)

where the id parameter specifies clustering at the school level; strata accounts for stratification by geographic and demographic characteristics within states; weights applies state-specific sampling weights, accounting for selection probabilities, non-response, and post-stratification; nest=TRUE allows PSU codes to be unique within strata. States function as independent sampling frames; PSUs nest within states and do not cross state boundaries, such that the design inherently accounts for both school- and state-level clustering.

Variance estimation used Taylor series linearization, providing design-consistent standard errors accounting for stratification, clustering, and weights. Survey-weighted logistic regression (svyglm) with quasi-binomial variance family accounted for overdispersion (dispersion parameter = 1.0003). Design effects were 2.86 (gun carrying) and 4.78 (suicide attempt), yielding effective sample sizes of 8,133 and 4,864, respectively (nominal n = 23,232).

State-specific sampling weights were provided by the CDC and designed to produce state-representative estimates. Weights account for (1) unequal selection probabilities (schools selected with probability proportional to size), (2) school and student non-response, and (3) post-stratification to match state population totals for grade and sex.

eMethods 4. Ecological Policy Variable Treatment

State gun law scores represent an ecological (state-level) variable measured at a higher level of aggregation than the individual-level exposure and outcome. To address potential ecological fallacy and facilitate interpretation, we grand-mean centered the score variable (mean = 28.14) and scaled effects to per-10-unit increases. Models including state gun law score were specified as: suicide ~ gun + race + score_per10, where score_per10 represents 10-point increments on the 0-100 scale.

To assess state-level confounding, we conducted sensitivity analyses comparing models with and without state fixed effects. The baseline model included state gun law score as a continuous predictor, while the sensitivity model replaced the score with state indicator variables (factor(state)), removing between-state variation and estimating the gun carrying-suicide association solely from within-state comparisons. Minimal change in the gun carrying OR between models (< 1 percent) indicates the association is robust to unmeasured state-level confounding. States were not treated as an additional level in a multilevel model because our survey design already accounts for clustering within states through stratification and the nest=TRUE specification, with state-specific sampling weights producing state-representative estimates.

eMethods 5. Confounding Control Strategy

We adjusted for demographic characteristics (race/ethnicity, with sensitivity analyses adding sex and grade) to estimate the total association between gun carrying and suicide attempts before examining mechanistic pathways. Many variables associated with both outcomes (e.g., depression, bullying, substance use) may function as mediators (on causal pathways) rather than confounders. Adjusting for mediators in primary models induces over-adjustment bias, blocking pathways and underestimating

© 2026 Lindsey MA et al. *JAMA Network Open*.

total effects. We plan formal mediation analyses in subsequent papers using appropriate causal inference methods.

A directed acyclic graph (DAG) guided covariate selection. Gun carrying and suicide attempts share potential confounders (demographic characteristics including sex, grade, and race/ethnicity) and potential mediators (depression, substance use, bullying victimization, violence exposure). The distinction between confounders and mediators has critical implications for model specification:

Confounders (adjusted in primary models): Sex, grade, and race/ethnicity are pre-existing demographic characteristics that may influence both gun carrying behavior and suicide attempt risk but are not caused by gun carrying. Adjustment for these variables reduces confounding bias.

Potential mediators (not adjusted in primary models): Depression (persistent sadness), substance use, bullying victimization, and violence exposure may lie on the causal pathway between gun carrying and suicide attempts. For example, gun carrying may increase violence exposure, which in turn increases suicide risk. Adjusting for mediators in the primary model would induce over-adjustment bias, blocking the very pathways through which gun carrying may influence suicide risk and producing a biased underestimate of the total effect (VanderWeele TJ. Principles of confounder selection. Eur J Epidemiol. 2019;34:211-219).

Sensitivity analyses adding sex and grade to race-adjusted models yielded stronger estimates (OR 3.40 vs 4.51; 32.6 percent increase), demonstrating that the association persists after demographic adjustment. The increase in effect size after adjustment for sex and grade suggests negative confounding, where demographic factors suppress rather than explain the association. This pattern is consistent with sex differences in both gun carrying (higher among males) and suicide attempts (higher among females), which counteract each other in unadjusted analyses.

eTable 1. Missing Data Patterns and Characteristics

Pattern	N	% of Total	Description
Complete cases	23,232	80.8	No missingness on key variables
Missing suicide only	~2,200	~7.6	Missing suicide attempt item only
Missing gun only	~900	~3.1	Missing gun carrying item only
Missing both	~600	~2.1	Missing both exposure and outcome
Other patterns	~1,650	~5.8	Missing race, age, or grade combinations
**Total**	**28,765**	**100**	All students from 15 included states
**Little's MCAR test:** chi-squared = 1210.12, df = 58, P < .001, MCAR rejected. Patterns support MAR conditional on observed covariates.<br>**Note:** N and percentages are unweighted counts. Missing data patterns analyzed prior to survey weighting.			

eTable 2. Multiple Imputation Diagnostics Summary

© 2026 Lindsey MA et al. *JAMA Network Open*.

Parameter	FMI	MC Error (% of SE)	Relative Efficiency	Assessment
Intercept	0.248	18.3	0.992	Moderate influence
Gun carrying	0.181	18.3	0.994	Moderate influence
Race: Black (vs Other)	0.176	18.3	0.994	Moderate influence
Race: Hispanic (vs Other)	0.228	18.3	0.992	Moderate influence
Race: White (vs Other)	0.212	18.3	0.993	Moderate influence
Race: Other (vs Other)	0.216	18.3	0.993	Moderate influence
**Interpretation:** FMI values 0.176 to 0.248 indicate moderate missing-data influence. With m = 30 imputations, relative efficiencies exceed 0.99, confirming stable estimates. Monte Carlo errors of 18.3 percent of SE are acceptable for m = 30; sensitivity checks at m = 60 yielded odds ratios within 2 percent (results unchanged).<br>**Note:** Statistics derived from pooled MI analysis (m = 30 datasets chosen a priori). FMI = fraction of missing information; MC Error = Monte Carlo error as percentage of SE; Relative Efficiency = efficiency relative to infinite imputations.				

eTable 3. Multiple Imputation Model Specification

Variable	Type	Imputation Method	Predictors	PSU/Strata/Weights Included
Suicide attempt	Binary	Logistic regression (logreg)	All variables + design	Yes (passive)
Gun carrying	Binary	Logistic regression (logreg)	All variables + design	Yes (passive)
Sex	Binary	Logistic regression (logreg)	All variables + design	Yes (passive)
Race/ethnicity	Polytomous (4 levels)	Multinomial logistic (polyreg)	All variables + design	Yes (passive)
Grade	Ordinal (4 levels)	Proportional odds model (polr)	All variables + design	Yes (passive)
Age	Continuous	Predictive mean matching (pmm)	All variables + design	Yes (passive)
PSU	Design variable	Not imputed	–	–
Strata	Design variable	Not imputed	–	–
Weight	Design variable	Not imputed	–	–
**Specification Details:** - **Software:** mice package version 3.18.0 in R 4.5.1 - **Number of imputations:** m = 30 - **Iterations:** 15 (convergence achieved by iteration 10) - **Imputation model:** Each variable imputed using all other variables as predictors, including PSU, strata, and weights as passive predictors - **Convergence:** Visual inspection of trace plots and Gelman-Rubin diagnostics				

© 2026 Lindsey MA et al. *JAMA Network Open*.

Variable	Type	Imputation Method	Predictors	PSU/Strata/Weights Included
confirmed convergence for all imputed variables - **Distribution matching:** Imputed values matched observed distributions (maximum difference less than 0.2 percent) - **Pooling:** Rubin's rules with Barnard-Rubin degrees of freedom adjustment<br>**Note:** Design variables (PSU, strata, weights) were included as predictors in imputation models but were not themselves imputed, preserving the survey design structure.				

eTable 4. Sensitivity Analysis: Complete Case vs Multiple Imputation

Method	N	Gun Carrying OR	95% CI	% Difference
Complete Case Analysis	23,232	3.72	2.82-4.90	Reference
Multiple Imputation (m = 30)	28,765	2.91	2.19-3.86	-21.8
**Interpretation:** Multiple imputation yielded a slightly lower estimate (OR 2.91) compared to complete case analysis (OR 3.72), representing a 21.8 percent difference. Both estimates remain highly significant and indicate a strong association between gun carrying and suicide attempts, confirming robustness despite modest attenuation under MI.<br>**Note:** N are unweighted counts. Odds ratios and confidence intervals are survey-weighted with Taylor series linearization.				

eTable 5. Characteristics of the 15 States Included in Analysis

State	Abbrev	N (Post-CCA)	Gun Law Score	Region	Gun Law Category
Arkansas	AR	582	3.0	South	Very Weak (<=12)
Mississippi	MS	434	3.0	South	Very Weak (<=12)
Montana	MT	3,182	5.0	West	Very Weak (<=12)
Kentucky	KY	1,664	9.0	South	Very Weak (<=12)
Utah	UT	687	12.0	West	Very Weak (<=12)
Texas	TX	877	13.5	South	Weak-Moderate (13-34)
Tennessee	TN	1,077	16.5	South	Weak-Moderate (13-34)
North Carolina	NC	1,991	25.0	South	Weak-Moderate (13-34)
Nebraska	NE	542	25.0	Midwest	Weak-Moderate (13-34)
Nevada	NV	1,211	35.0	West	Moderate-Strong (35-49)
Michigan	MI	1,899	35.0	Midwest	Moderate-Strong (35-49)
Pennsylvania	PA	1,825	40.0	Northeast	Moderate-Strong (35-49)
New Mexico	NM	4,997	40.5	West	Moderate-Strong (35-49)
Virginia	VA	1,776	49.0	South	Moderate-Strong (35-49)
New York (excl NYC)	NYA	488	83.5	Northeast	Very Strong (>=50)
**Total**	–	**23,232**	**Mean 23.7**	–	–

State	Abbrev	N (Post-CCA)	Gun Law Score	Region	Gun Law Category
**Summary:** Sample sizes range from 434 (Mississippi) to 4,997 (New Mexico). Gun law scores range from 3.0 (Arkansas, Mississippi) to 83.5 (New York). Eight states are from the South, four from the West, two from the Midwest, and two from the Northeast. Gun law strength categories: Very Weak (<=12): 5 states, n = 6,549; Weak-Moderate (13-34): 4 states, n = 4,487; Moderate-Strong (35-49): 5 states, n = 11,708; Very Strong (>=50): 1 state, n = 488.<br>**Note:** N (Post-CCA) are unweighted counts. CCA = complete case analysis. Gun law scores derived from Everytown for Gun Safety's 2023 Gun Law Rankings, 0 to 100 scale where higher indicates stricter policy (see eMethods 5). No survey weighting applied to this table.					

eTable 6. Design Effects and Effective Sample Sizes

Variable	Prevalence (%)	SE (Design)	SE (Naive)	DEFF	Effective N
Gun carrying	5.0	0.00226	0.00133	2.86	8,133
Suicide attempt	9.6	0.00434	0.00199	4.78	4,864
**Interpretation:** The survey design increases variance by 286 percent for gun carrying and 478 percent for suicide attempt relative to simple random sampling. These design effects were accounted for by Taylor series linearization. Effective sample sizes correspond to precision-adjusted n used in inference. The larger design effect for suicide attempt (4.78) compared to gun carrying (2.86) reflects higher intraclass correlation for mental health outcomes within schools.<br>**Note:** Prevalence values are survey-weighted and match pooled estimates in eTable 2 (gun carrying 5.0 percent, suicide attempts 9.6 percent). Standard errors use Taylor series linearization. DEFF = design effect = (SE_design / SE_naive)^2; Effective N = nominal N / DEFF.					

eTable 7. Sensitivity Analysis: State Fixed Effects vs Ecological Variable

Model	N	Covariates	Gun Carrying OR	95% CI	State Gun Law Score OR (per 10 units)	95% CI	Change from Baseline
Model 1: Baseline	23,232	Race + Score (continuous)	3.72	2.82-4.90	0.93	0.88-0.99	Reference
Model 2: State FE	23,232	Race + State indicators	3.75	2.80-5.01	–	–	+0.8%
**Interpretation:** Near-identical odds ratios indicate robustness to unmeasured state-level confounding. OR 0.93 per 10-point increase in gun law score indicates 6.7 percent lower odds of suicide attempt per 10-point gain. The minimal change (0.8 percent) when replacing the continuous ecological variable with state fixed effects demonstrates that the individual-level association between gun carrying and suicide attempts does not depend on state-level factors or between-state variation in gun laws.<br>**Note:** N are unweighted counts. Odds ratios and confidence intervals are survey-weighted with Taylor series linearization. FE = fixed effects. State fixed effects model could not include the continuous score variable due to collinearity.							

© 2026 Lindsey MA et al. *JAMA Network Open*.

eTable 8. Multiplicative Interaction Terms Between Gun Carrying and Race and Ethnicity and State Gun Law Strength

Interaction	Observed OR	95% CI	P Value
Gun x Hispanic (vs non-Hispanic Black)	1.38	0.56-3.38	.472
Gun x non-Hispanic White	0.89	0.39-2.05	.783
Gun x Other race	1.00	0.43-2.32	.999
Gun x State law score (per 10 points)	1.02	1.00-1.04	.114
Gun x State law category (weak vs strong)	1.13	0.60-2.12	.698
**Interpretation:** None of the tested interactions reached statistical significance at α = .05. The 95% confidence intervals are wide and do not exclude moderate effect modification in either direction; the data are therefore compatible with a range of true effect-modification magnitudes and these findings are best characterized as inconclusive rather than as evidence against effect modification. **Note:** Observed ORs and 95% confidence intervals are survey-weighted with Taylor series linearization variance estimation.			

eTable 9. Sex-Stratified Gun Carrying and Suicide Attempt Prevalence

Characteristic	Male	Female
N (unweighted)	11,241	11,886
Weighted % of sample	49.0%	50.5%
Gun carrying prevalence	7.9% (889/11,241)	2.2% (262/11,886)
% of gun carriers (weighted)	74.7%	24.4%
% of non-carriers (weighted)	47.9%	51.6%
Suicide attempt prevalence (overall)	Available from study data	Available from study data
SA prevalence among gun carriers	Available from study data	Available from study data
SA prevalence among non-carriers	Available from study data	Available from study data
**Key Patterns:** - Males are dramatically overrepresented among gun carriers (74.7 percent of carriers vs 49.0 percent of the overall sample) - Females are underrepresented among carriers (24.4 percent of carriers vs 50.5 percent of the overall sample) despite higher overall suicide attempt rates - Among non-carriers, the sex distribution closely mirrors the overall sample (47.9 percent male, 51.6 percent female) - This opposing pattern (males more likely to carry guns, females more likely to attempt suicide) produces negative confounding in unadjusted analyses, explaining the 32.6 percent increase in OR after sex adjustment (eTable 10)<br>**Note:** N are unweighted counts. Percentages are survey-weighted. SA = suicide attempt. Sex-stratified suicide attempt prevalences by carrier status are available from study data but are not reported in this table to avoid small cell concerns. The remaining 0.5 percent of the weighted sample and 1.0 percent of carriers represent students with missing or other sex responses. Approximately 105 students had missing sex data and 10 of those were gun carriers.		

State Participation and Exclusion Criteria, 2023 YRBS

© 2026 Lindsey MA et al. *JAMA Network Open*.

State	Code	Status
Arkansas	AR	Included in analysis
Kentucky	KY	Included in analysis
Michigan	MI	Included in analysis
Mississippi	MS	Included in analysis
Montana	MT	Included in analysis
Nebraska	NE	Included in analysis
Nevada	NV	Included in analysis
New Mexico	NM	Included in analysis
New York (excl NYC)	NYA	Included in analysis
North Carolina	NC	Included in analysis
Pennsylvania	PA	Included in analysis
Tennessee	TN	Included in analysis
Texas	TX	Included in analysis
Utah	UT	Included in analysis
Virginia	VA	Included in analysis
**Missouri**	**MO**	**Excluded: 100 percent missing suicide attempt data**
Alabama	AL	Excluded: did not collect gun carrying data
California	CA	Excluded: did not collect gun carrying data
Connecticut	CT	Excluded: did not collect gun carrying data
Delaware	DE	Excluded: did not collect gun carrying data
Hawaii	HI	Excluded: did not collect gun carrying data
Idaho	ID	Excluded: did not collect gun carrying data
Illinois	IL	Excluded: did not collect gun carrying data
Maine	ME	Excluded: did not collect gun carrying data
Maryland	MD	Excluded: did not collect gun carrying data
Rhode Island	RI	Excluded: did not collect gun carrying data
Vermont	VT	Excluded: did not collect gun carrying data
West Virginia	WV	Excluded: did not collect gun carrying data
**Summary:** Total participating states = 28; included = 15; excluded = 13. Twelve states did not collect the firearm item; Missouri had 100 percent missing suicide data.<br>**Note:** This table documents state participation status; no weighting applied. State codes match YRBS dataset conventions (NYA = New York State excluding New York City).		

Weight Distribution by State

State	N	Mean Weight	Median	Min	Max	CV	Eff N
AR	582	211.5	172.0	90.5	930.9	0.650	409

© 2026 Lindsey MA et al. *JAMA Network Open*.

State	N	Mean Weight	Median	Min	Max	CV	Eff N
KY	1,664	103.0	95.8	44.5	413.0	0.339	1,492
MI	1,899	185.1	164.2	44.0	602.2	0.464	1,563
MS	434	219.9	173.0	56.9	744.0	0.659	303
MT	3,182	12.6	12.0	2.8	30.0	0.408	2,728
NC	1,991	207.9	171.0	30.5	734.5	0.652	1,397
NE	542	168.3	133.4	53.1	1,234.0	0.724	356
NM	4,997	15.0	13.9	2.8	49.0	0.431	4,214
NV	1,211	89.2	84.4	38.4	214.8	0.352	1,077
NYA	488	740.5	709.2	230.2	1,954.9	0.476	398
PA	1,825	250.2	238.5	43.2	651.7	0.384	1,590
TN	1,077	197.3	163.6	16.7	650.6	0.607	787
TX	877	1,648.1	1,467.2	769.9	5,266.1	0.421	745
UT	687	271.5	221.0	150.2	751.6	0.403	591
VA	1,776	206.3	188.2	61.0	559.0	0.477	1,446
**Pooled**	**23,232**	**193.6**	**103.7**	**2.8**	**5,266.1**	**1.833**	**5,329**

Note: N are unweighted counts. Weight moments (mean, median, min, max, CV) are computed over individual student records. CV = coefficient of variation (SD / mean). Effective N = (sum of w)^2 / sum of w^2, representing precision-adjusted sample size accounting for unequal weighting. State-specific sampling weights produce state-representative estimates and account for unequal selection probabilities, nonresponse, and post-stratification.

eTable 10. Characteristics by Gun Law Strength Category

Characteristic	Very Weak (<=12)	Weak-Moderate (13-34)	Moderate-Strong (35-49)	Very Strong (>=50)
N states	5	4	5	1
N students	6,549	4,487	11,708	488
States included	AR, MS, MT, KY, UT	TX, TN, NC, NE	NV, MI, PA, NM, VA	NYA
Gun law score range	3.0-12.0	13.5-25.0	35.0-49.0	83.5
Gun carrying prevalence	7.0%	5.5%	3.9%	2.9%
Suicide attempt prevalence	10.1%	10.5%	9.2%	7.8%

Note: N are unweighted counts. Prevalences are survey-weighted. Gun law categories based on Everytown for Gun Safety 2023 Gun Law Strength Scores (see eMethods 5).

eTable 11. State-Level Weighted Prevalences

State	N	Gun Carrying Prevalence (%)	95% CI	Suicide Attempt Prevalence (%)	95% CI	DEFF (Gun)	DEFF (Suicide)
AR	582	7.8	4.9-12.2	11.2	7.8-15.9	2.1	3.2
KY	1,664	6.2	4.7-8.1	10.8	8.9-13.0	2.4	4.1
MI	1,899	3.9	2.8-5.4	9.1	7.5-11.0	2.8	4.9
MS	434	8.1	5.2-12.4	12.4	8.9-17.0	1.9	3.1

State	N	Gun Carrying Prevalence (%)	95% CI	Suicide Attempt Prevalence (%)	95% CI	DEFF (Gun)	DEFF (Suicide)
MT	3,182	5.3	4.2-6.6	8.9	7.6-10.4	3.1	5.2
NC	1,991	4.7	3.5-6.2	10.2	8.6-12.1	2.6	4.5
NE	542	5.9	3.9-8.9	9.8	7.2-13.2	2.2	3.8
NM	4,997	4.1	3.4-4.9	9.4	8.4-10.5	2.9	5.1
NV	1,211	3.8	2.6-5.5	8.7	7.0-10.8	2.5	4.3
NYA	488	2.9	1.6-5.1	7.8	5.4-11.1	2.1	3.6
PA	1,825	4.2	3.1-5.7	9.5	8.0-11.3	2.7	4.7
TN	1,077	6.8	5.1-9.0	11.1	8.9-13.8	2.3	3.9
TX	877	5.4	3.9-7.5	10.3	8.2-12.9	2.6	4.4
UT	687	4.5	3.0-6.7	8.2	6.1-10.9	2.2	3.7
VA	1,776	4.6	3.4-6.2	9.7	8.1-11.6	2.7	4.6
**Pooled**	**23,232**	**5.0**	**4.2-5.9**	**9.6**	**8.8-10.5**	**2.86**	**4.78**
**Summary:** State-level weighted prevalences show heterogeneity in both gun carrying (range: 2.9 percent to 8.1 percent) and suicide attempts (range: 7.8 percent to 12.4 percent). Design effects vary by state, reflecting differences in clustering and sample design. Pooled estimates reflect survey-weighted combination across all 15 state designs; standard errors via Taylor series linearization. **Note:** Prevalences are survey-weighted; confidence intervals computed using Taylor series linearization on the identity scale. N are unweighted counts. DEFF = design effect = (SE_design / SE_SRS)^2.							

eTable 12. Race-Stratified Cell Counts and Suicide Attempt Prevalences

Race/Ethnicity	Total N	Gun Carriers	Non-Carriers	SA Among Carriers	SA Among Non-Carriers	No SA Among Carriers	No SA Among Non-Carriers
Black	2,262	108	2,154	44 (35.2%)	255 (14.2%)	64	1,899
Hispanic	6,929	361	6,568	95 (30.5%)	554 (9.2%)	266	6,014
White	10,815	487	10,328	89 (21.3%)	819 (7.9%)	398	9,509
Other	3,226	205	3,021	56 (30.9%)	324 (11.1%)	149	2,697
**Total**	**23,232**	**1,161**	**22,071**	**284 (27.4%)**	**1,952 (9.4%)**	**877**	**20,119**
Race-Stratified Risk Ratios:							

© 2026 Lindsey MA et al. *JAMA Network Open*.

Race/Ethnicity	Total N	Gun Carriers	Non-Carriers	SA Among Carriers	SA Among Non-Carriers	No SA Among Carriers	No SA Among Non-Carriers

Race/Ethnicity	N	Carriers (N)	SA Prevalence Among Carriers	SA Prevalence Among Non-Carriers	Risk Ratio	95% CI
Black	2,262	108	35.2%	14.2%	2.49	1.49-4.15
Hispanic	6,929	361	30.5%	9.2%	3.31	2.05-5.35
White	10,815	487	21.3%	7.9%	2.68	1.92-3.75
Other	3,226	205	30.9%	11.1%	2.78	1.79-4.33
**Overall**	**23,232**	**1,161**	**27.4%**	**9.4%**	**2.91**	**2.35-3.60**

Race-Stratified Odds Ratios (survey-weighted logistic regression):

Race/Ethnicity	N	OR	95% CI	P Value
Black	2,262	3.30	1.56-6.95	.003
Hispanic	6,929	4.33	2.25-8.36	< .001
White	10,815	3.14	2.02-4.88	< .001
Other	3,226	3.58	2.15-5.97	< .001

Note: N are unweighted counts. SA = suicide attempt. SA prevalence percentages among carriers and non-carriers are survey-weighted. Risk ratios and odds ratios are from survey-weighted analyses with Taylor series linearization. Racial/ethnic categories are ordered alphabetically; Other includes Asian, Native American, Pacific Islander, and multiracial students. Black = non-Hispanic Black; White = non-Hispanic White. The reference group for within-group comparisons is non-carriers within each racial/ethnic group.

eTable 13. Sensitivity Analysis: Demographic Confounding (Sex and Grade)

Model	N	Covariates	Gun Carrying OR	95% CI	Change from Baseline
Model 1: Baseline	23,232	Race/ethnicity	3.40	2.55-4.53	Reference
Model 2: Adjusted	23,232	Race/ethnicity + Sex + Grade	4.51	3.24-6.26	+32.6%

Interpretation: The gun carrying association strengthens by 32.6 percent after adjusting for sex and grade, consistent with negative confounding due to opposing sex differences in exposure and outcome. Males carry guns at substantially higher rates than females (74.7 percent vs 24.4 percent of carriers), while females attempt suicide at higher rates. In unadjusted analyses, these opposing patterns counteract each other, yielding conservative estimates. After adjusting for sex, the association within sex strata emerges more clearly, revealing a stronger relationship.
Note: N are unweighted counts. Odds ratios and confidence intervals are survey-weighted with Taylor series linearization.

eFigure. Participant Flow Diagram (CONSORT Style)

State-Level Exclusions

© 2026 Lindsey MA et al. *JAMA Network Open*.

28 states participated in 2023 YRBS
|
|---> 12 states excluded: did not collect firearm carrying data
|
|---> 1 state excluded: Missouri (100% missing suicide attempt data)
|
|---> 15 states included in analysis (n = 28,765 students)

Student-Level Exclusions (Complete Case Analysis)
28,765 students from 15 states
|
|---> Missing race/ethnicity: 555 (1.93%)
|---> Missing gun carrying: 1,278 (4.44%)
|---> Missing suicide attempts: 4,118 (14.32%)
|---> Final analytic sample: 23,232 students (80.8% retention)

Stage	N States	N Students	Excluded N	% Retained (Cumulative)
States in 2023 YRBS	28	–	–	–
Excluded: no firearm data	(12)	–	–	–
Excluded: Missouri	(1)	–	–	–
**Included states**	**15**	**28,765**	–	**100.0**
Missing race/ethnicity	–	28,210	555	98.1
Missing gun carrying	–	27,487	1,278	95.6
Missing suicide attempt	–	24,647	4,118	85.7
**Final analytic sample**	**15**	**23,232**	**5,533**	**80.8**

Note: Exclusion categories are not mutually exclusive; students may be missing multiple variables. Percent retained is cumulative retention relative to the 28,765 students from included states. Total excluded N = 5,533 represents unique students with any missing data on race/ethnicity, gun carrying, or suicide attempts.

eResults. Summary of Supplementary Findings

1. State heterogeneity (eTables 1, 2): The 15 included states represent diverse geographic regions and gun law environments. State-level prevalences show substantial heterogeneity in both gun carrying (2.9 percent to 8.1 percent) and suicide attempts (7.8 percent to 12.4 percent), supporting the importance of state-representative (rather than nationally representative) estimates.
2. Design and weights (eTable 5): Design effects of 2.86 (gun carrying) and 4.78 (suicide attempts) substantially reduce effective sample sizes, confirming the necessity of survey-weighted analysis with Taylor series linearization for valid inference.
3. Missing data (eTables 6-9): Little's MCAR test rejected ($P < .001$), but patterns support MAR conditional on observed covariates. Multiple imputation with m = 30 imputations yielded adequate relative efficiency (>0.99) and moderate FMI (0.18-0.25). Complete case analysis (OR 3.72) and MI (OR 2.91) both show strong associations, confirming robustness despite 21.8 percent attenuation under MI.

© 2026 Lindsey MA et al. *JAMA Network Open*.

4. Confounding and robustness (eTables 10, 11): Adding sex and grade strengthened the association by 32.6 percent (OR 3.40 to 4.51), indicating negative confounding. State fixed effects analysis showed minimal change (< 1 percent), demonstrating robustness to unmeasured state-level confounding.
5. Interaction tests (eTable 12): No interaction term between gun carrying and race/ethnicity or state gun law strength reached statistical significance. Confidence intervals were wide and do not exclude clinically meaningful effect modification in either direction; these findings are characterized as inconclusive.
6. Sex-stratified patterns (eTable 13): Males are dramatically overrepresented among gun carriers (74.7 percent) relative to their share of the sample (49.0 percent), while females are underrepresented among carriers (24.4 percent) despite higher suicide attempt rates. This opposing pattern produces the negative confounding observed in sensitivity analyses.
7. Race-stratified associations (eTable 3): Gun carrying was associated with elevated suicide attempt risk across all racial/ethnic groups, with risk ratios ranging from 2.49 (Black) to 3.31 (Hispanic). Racial categories are presented in alphabetical order: Black, Hispanic, White, Other.

Abbreviations

aOR, adjusted odds ratio; CCA, complete case analysis; CI, confidence interval; CV, coefficient of variation; DAG, directed acyclic graph; DEFF, design effect; FE, fixed effects; FMI, fraction of missing information; logreg, logistic regression; MAR, missing at random; MCAR, missing completely at random; MI, multiple imputation; OR, odds ratio; pmm, predictive mean matching; polr, proportional odds logistic regression; polyreg, polytomous logistic regression; PSU, primary sampling unit; RR, risk ratio; SA, suicide attempt; SE, standard error; YRBS, Youth Risk Behavior Survey.

eReferences

1. Lumley T. Analysis of Complex Survey Samples. *J Stat Soft*. 2004;9(8). doi:10.18637/jss.v009.i08
2. Rubin DB. *Multiple Imputation for Nonresponse in Surveys*. 1st ed. Wiley; 1987. doi:10.1002/9780470316696
3. Skinner CJ, Holmes DJ. Random Effects Models for Longitudinal Survey Data. In: Chambers RL, Skinner CJ, eds. *Analysis of Survey Data*. 1st ed. Wiley; 2003:205-219. doi:10.1002/0470867205.ch14
4. Heeringa S, West BT, Berglund PA. *Applied Survey Data Analysis*. Second edition. CRC Press, Taylor & Francis Group; 2017.
5. VanderWeele TJ. Principles of confounder selection. *Eur J Epidemiol*. 2019;34(3):211-219. doi:10.1007/s10654-019-00494-6
6. Everytown for Gun Safety Support Fund. *Everytown Gun Law Rankings*. Everytown for Gun Safety Support Fund; 2023.
7. van Buuren SV, Groothuis-Oudshoorn K. **mice** : Multivariate Imputation by Chained Equations in *R*. *J Stat Soft*. 2011;45(3):1-67. doi:10.18637/jss.v045.i03

© 2026 Lindsey MA et al. *JAMA Network Open*.
