# Supplementary material for: Carrying Firearms and Suicide Risk Among Adolescents
Source: JAMA Netw Open. 2026 Aug 5;9(8):e2626924. doi: 10.1001/jamanetworkopen.2026.26924 (PMC13444804; doi:10.1001/jamanetworkopen.2026.26924)
Supplement: Supplementary file 2 [file jamanetwopen-e2626924-s002.pdf]

Data Sharing Statement

Lindsey. Carrying Firearms and Suicide Risk Among Adolescents. *JAMA Netw Open*. Published August 05, 2026. doi:10.1001/jamanetworkopen.2026.26924

Data
Data available: Yes
Data types: Deidentified participant data
How to access data: https://www.cdc.gov/yrbs/index.html
When available: With publication

Supporting Documents
Document types: Statistical/analytic code
How to access documents: Email: Michael.Lindsey@nyu.edu
When available: With publication

Additional Information
Who can access the data: Anyone requesting the data
Types of analyses: For any purpose
Mechanisms of data availability: With investigator support
